# Application of culture, PCR, and PacBio sequencing for determination of microbial composition of milk from subclinical mastitis dairy cows of smallholder farms

**DOI:** 10.1515/biol-2021-0080

**Published:** 2021-08-18

**Authors:** Ntelekwane G. Khasapane, Jane S. Nkhebenyane, Stanford Kwenda, Zamantungwa T. H. Khumalo, Phillip S. Mtshali, Moeti O. Taioe, Oriel M. M. Thekisoe

**Affiliations:** Department of Life Sciences, Centre for Applied Food Sustainability and Biotechnology, Central University of Technology, Bloemfontein, 9300, South Africa; Sequencing Core Facility, National Institute for Communicable Diseases, National Health Laboratory Service, Johannesburg, South Africa; Epidemiology, Parasites and Vectors, Agricultural Research Council – Onderstepoort Veterinary Research (ARC-OVR), Onderstepoort 0110, South Africa; Unit for Environmental Sciences and Management, North-West University, Potchefstroom, 2531, South Africa; Department of Veterinary Tropical Diseases, Faculty of Veterinary Science, University of Pretoria, Private Bag X04, Onderstepoort, 0110, South Africa

**Keywords:** microbiota, mastitis, 16S ribosomal RNA, DNA sequencing, microbial milk composition

## Abstract

Mastitis is a cow disease usually signalized by irritation, swelling, and soreness of the udder. It is characterized by physical, chemical, and biological changes in the udder and milk. The aim of this study was to detect and characterize pathogens causing subclinical mastitis (SCM) from the milk of dairy cows of small-scale farmers through culture and molecular techniques. Milk was collected from 32 cows belonging to 8 small-scale farmers around Harrismith District, South Africa. The results showed that screening of SCM by California mastitis test and somatic cell counts (SCC) was 21.87 and 25%, respectively. Culture methods revealed the presence of *Staphylococcus aureus* at 93% followed by *Streptococci* spp. and *Escherichia coli* at 36.4 and 13.3%, respectively. The PCR could only detect *E. coli*, while single-molecule real-time sequencing showed a total of 2 phyla, 5 families, 7 genera, and 131 species. Clostridiaceae was the most abundant family, while *Romboutsia* was the most abundant genus followed by *Turicibacter* spp. The present study has documented the occurrence of SCM causing pathogens in milk collected from cows of small-scale farmers in Harrismith, indicating that SCM may be present at higher levels than expected.

## 1 Introduction

Mastitis is a cow infection characterized by inflammation of the teats [1]. Mastitis in bovines has been regarded as a major economic drain in the dairy sector worldwide [2]. Furthermore, the economic burden of this infection manifests in factors such as low milk production during pre- and post-infection, the need to administer medicinal agents, low fertility rates, and the onset of the culling of bovines [3]. Mastitis also affects the vital nutrients in milk which leads to reduced nutrient quantities [4]. This infection is categorized as clinical mastitis or subclinical mastitis (SCM), with the former being observed when the inflammatory response is robust and causes visible modifications in the milk (e.g., clots and color changes), a swollen udder, and symptoms of ill health displayed by the cow (e.g., off-feed and dehydration) [5]. SCM has asymptomatic characteristics and hence, the need to screen bovines for infection through somatic cell counts (SCC) and California mastitis test (CMT) [6].

The sudden onset of this infection in bovines is due to bacterial, mycotic, algal, and, in some instances, viral species attacking the tissue surrounding the udder, which results in the inflammation of the mammary glands [7]. It has been shown that factors such as inadequate sanitation of the milking shed and deprived animal health services play a role in the development and duration of this infection. So far, about 135 microbial strains have been identified as causal agents of mastitis in bovines, with *Streptococcus* spp. and *Staphylococcus* spp. being the most prevalent. Additionally, it has been observed that *Escherichia coli*, *Mycoplasma bovis*, and *Klebsiella pneumoniae* also cause mastitis in bovines [6].

Previously, the identification of mastitis-causing pathogens relied on time-consuming conventional methods, a period of at least 48 h was required to make a diagnosis, which prolonged the administration of treatment [8]. Therefore, to bypass the difficulties related to conventional methods for diagnosis and identification, DNA-based techniques are currently utilized to focus on the DNA composition of microorganisms instead of the colony phenotypic expression [9]. This is advantageous because identifying a pathogen is determined early and rapidly, allowing producers to devise rapid solutions and provides farmers an opportunity to promptly heal ill cows and return them back to the producing line [10]. In this study, CMT, SCC, bacteriological, high-throughput next generation sequencing as well as multiplex PCR were employed to detect and investigate SCM-causing agents in Harrismith, Maluti-A-Phofung Local Municipality, South Africa.

## 2 Materials and methods

### 2.1 Sample collection

**Figure 1:**
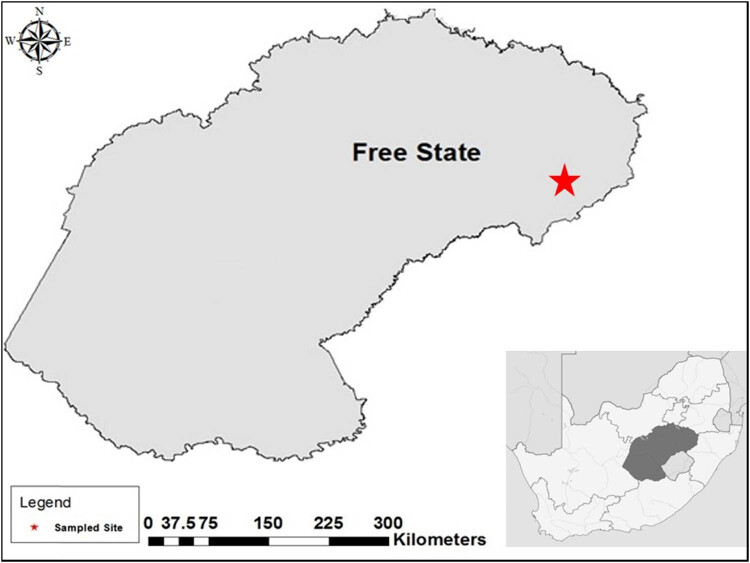
Free state province map of South Africa. The star indicates the sampled area in Harrismith Maluti-A-Phofung Local Municipality.

Milk samples were collected randomly from a total of 32 cows with asymptomatic teats at eight small-scale farms around Harrismith (Latitude: 28°16′21.94″S Longitude: 29°07′46.06″E) in Maluti-A-Phofung Local Municipality, Free State Province (Figure 1). The samples were collected using 50 mL sterile bottles and were transported to the laboratory (Centre for Applied Food Sustainability and Biotechnology) and analyzed within 6–8 h after sampling. During transportation, the samples were stored in a cooler box maintained at 4–6°C.

**Ethical approval:** The study was approved by the scientific committee of the Centre for Applied Food Safety and Biotechnology, Central University of Technology and the Department of Agriculture, Forestry and Fisheries for issuing section 20 permit (12/11/1/4/3) of the Animal Diseases Act (Act 35 of 1984). The state veterinarian in Harrismith, Dr Mukelabai Mundia and small-holder farmers were informed and further agreed to participate in this study.

### 2.2 Screening of cows

A California Mastitis Kit (DeLaval, South Africa) was used to assess whether the selected individual cows had intramammary infections (IMI) and thereby to determine SCM. After that, milk samples were sent to an outsourced laboratory for SCC (Swift Silliker (Pty) Ltd t/a Mérieux NutriSciences, Midrand, South Africa).

### 2.3 Microbiological analyses

For the isolation of various targeted microorganisms, 0.1 mL of diluted samples were plated out on blood agar (Oxoid, Thermo Fisher, UK), Violet red bile agar (Oxoid, Thermo Fisher, UK), and Baired Parker agar (Merck, SA) for enumeration of *Streptococcus* species, *Escherichia coli,* and *Staphylococcus aureus*. Subsequently, the plates were incubated at 35°C for 48 h, 35°C for 24–48 h, and 35°C for 24 h for *Streptococcus* species*, E. coli,* and *S. aureus,* respectively. After that, for preliminary counting and identification based on morphology and phenotypic characteristics, all plates were enumerated under the Interscience plate counter (78860, Saint Nom, France). Subsequently, all isolated colonies were subjected to gram staining and catalase test prior to the use of RapID identification kit and Staphylase test (Thermo Fisher, USA) for final confirmation of *E. coli, Streptococcus* spp., and *S. aureus* following manufacturer’s instructions.

### 2.4 DNA extraction

Genomic DNA was extracted using QIAamp DNA Mini Kit as per the manufacturer’s instructions (Qiagen, Hilden, Germany) with minor modifications before digestion. Briefly, 1 mL of the raw milk sample was inoculated in 9 mL of liquid media (nutrient broth) and incubated at 37°C for 24 h to obtain enough bacterial cells for analysis. After the 24 h period, a maximum of 5 × 10^6^ cells was centrifuged at 190 rpm for 5 min. Then, the cells were resuspended in 200 μL of phosphate-buffered saline, and 20 μL of proteinase K was then added to the mixture. Subsequently, the extraction protocol was followed to detail from the manufacturer’s instructions. The quality of the extracted DNA was assessed using 1.5% (w/v) of agarose gel stained with ethidium bromide and visualized under UV light. Finally, the DNA samples were frozen until needed.

### 2.5 Multiplex PCR

The primers that were used for the amplification of different pathogens through mPCR targeting the 16S rRNA of Sdys (*S. dysgalactiae*), sip (*S. agalactiae*), pau (*S. uberis*), *nuc* (*S. aureus*), and *alr* (*E. coli*) had product sizes of 549, 266, 439, 181, and 366 bp, respectively, as published in ref. [11].

### 2.6 Standard multiplex PCR (mPCR)

Standard mPCR was conducted using NEB OneTaq 2× MasterMix with Standard Buffer (10 μL). The reaction mixture contained gDNA (10–30 ng μL^−1^) (1 μL), forward primer (10 μM) (1 μL), reverse primer (10 μM) (1 μL), and nuclease-free water (7 μL). The reaction mixture was then mixed thoroughly by pipetting the mixture a few times, and subsequently, 20 μL of the final reaction mixture was dispensed into the PCR tubes. The PCR tubes were then placed in a thermal cycler for 35 cycles as follows: for the initial activation step, the tubes were subjected to 94°C for 5 min, denaturing occurred for 30 s at 94°C, annealing occurred for 30 s at 50°C, and extension occurred for 60 s at 68°C. The final extension was at 68°C for 10 min and the holding was at 4°C. After that, PCR amplicons were visualized on 1% of agarose gel (CSL-AG500, Cleaver Scientific [Ltd]) and stained with EZ-vision® Bluelight DNA dye under UV light.

### 2.7 PacBio sequencing

The diversity of bacterial communities in milk samples from various farms were analyzed using single-molecule real-time (SMRT) PacBio sequencing technology (Pacific Biosciences, Menlo Park, CA, USA). SMRTbell libraries were created using SMRTbell^™^ Template Prep Kit 1.0 (Pacific Biosciences, CA, USA) following instructions in the protocol “Procedure & Checklist – Amplicon Template Preparation and Sequencing” (part number 100-801-600-04). Sequencing was done using the Sequel^®^ Sequencing Kit 2.1 (Pacific Biosciences, CA, USA) with on-plate loading concentration of 4 pM.

### 2.8 DADA2 analysis

The DADA2 analysis workflow [13] implemented in R software package was used to analyze raw amplicon sequencing data generated using the PacBio Sequel System (Pacific Biosciences, CA, USA). To infer amplicon sequence variants, error-model learning and chimera removal were performed on the filtered reads using default DADA2 parameters. Taxonomic assignments were made based on the curated SILVA 16S rRNA database [16]. Taxa and abundance tables generated by DADA2 were imported into the phyloseq package v1.28.0 [12,14] for downstream analysis and visualization, including estimation of richness and visualization of the alpha-diversity, as well as visualization of differences in taxa abundance between the three samples under study. A basic phylogenetic tree was plotted using the package ape version 5.3 [15]. Furthermore, analysis of variance (ANOVA) was carried out to determine significant differences in the bacterial diversity on a Microsoft excel (Office 16).

## 3 Results

### 3.1 Screening of cows

The CMT was used to diagnose the first four cows from the selected eight farms in Harrismith, Maluti-A-Phofung Local Municipality. The results indicate that out of the 32 cows that were screened only 7 (21.87%) tested positive for CMT. While on the other hand, SCC results indicated that from 16 samples that were selected, 10 (62.5%) had SCCs ranging from 1 × 10^5^ to 5 × 10^5^ cells mL^−1^; 5 samples (31.25%) had SCCs of more than 5 × 10^5^ cells mL^−1^; and 1 sample (6.25%) had a SCC above the designated threshold. Moreover, of all the samples, only four (25%) had SCCs ranging from 1 × 10^5^ to 2 × 10^5^ cells^−1^. Therefore it was concluded that the prevalence of SCM in the cows of small-scale farmers in the study area was 25%.

### 3.2 Microbiological analysis

For the isolation and characterization of microorganisms, 16 of the milk samples were subjected to various standard phenotypical and biochemical methods. The isolates were identified at the genus level based on the size, shape, and color of the colony in question using the Interscience plate. The tests revealed that there were 40 isolates in total: presumptive *Staphylococcus* spp. (14); *E. coli* (15); and *Streptococcus* (11). A RapID identification kit and a staphylase test were also used to identify organisms at species level. The results further showed that *S. aureus* was the most abundant pathogen at 93%, followed by *Streptococcus* spp. at 36.4%, and *E. coli* at 14.3%.

### 3.3 Multiplex polymerase chain reaction (mPCR)

For the purpose of this study, 16 milk bacterial DNA were analyzed using mPCR to simultaneously detect the five most predominantly observed mastitis-causing pathogens, namely *E. coli, S. aureus*, *Strep. agalactiae, Strep. dysgalactiae*, and *Strep. uberis*. DNA was extracted directly from all the collected samples, irrespective of whether the samples had tested positive or negative for the CMT and SCC techniques. The results showed that mPCR could detect only *E. coli* (i.e., the *alr* gene) (Figure S1).

### 3.4 Microbial diversity

**Figure 2:**
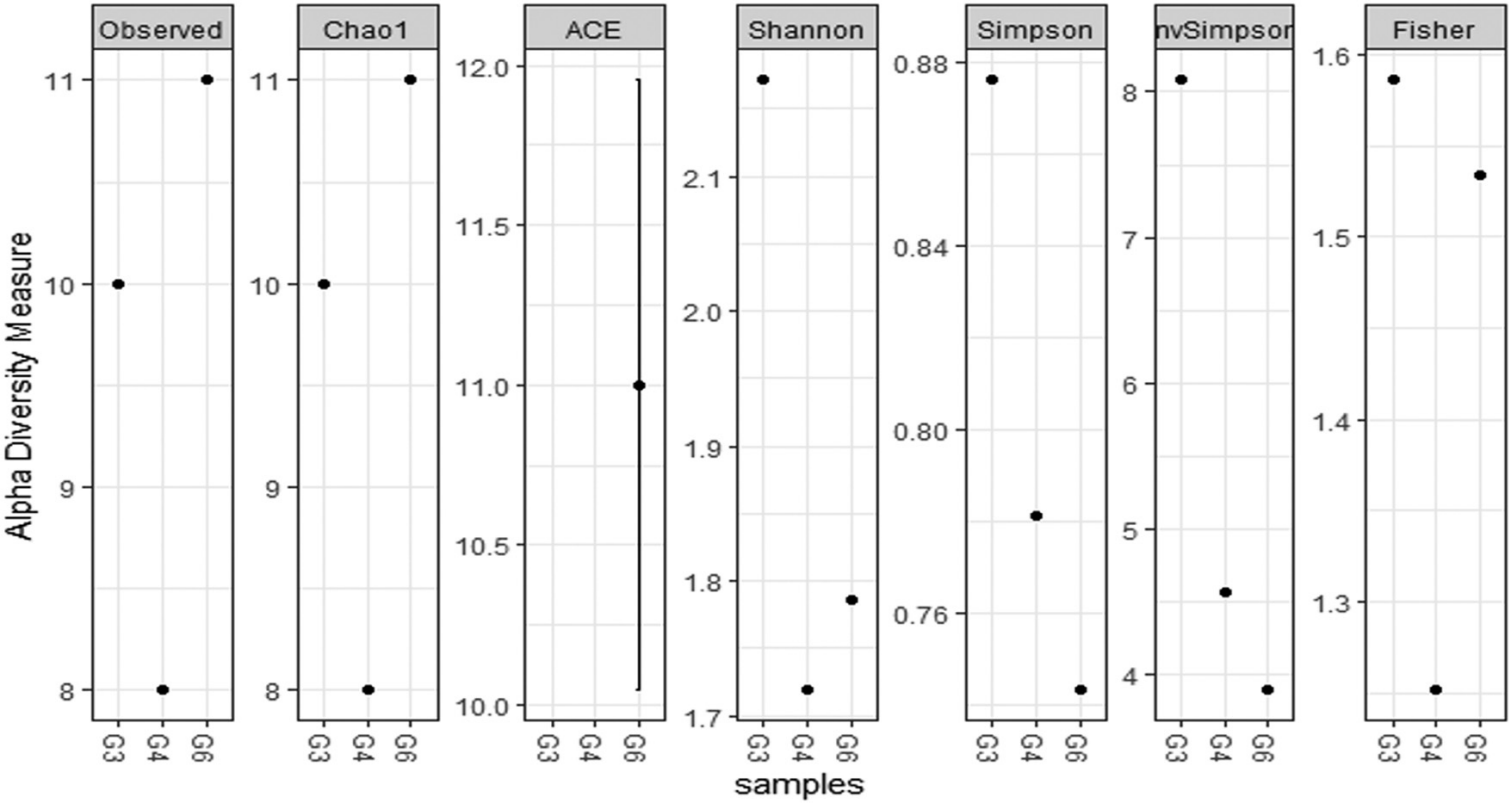
The comparison of richness plot from samples collected.

We performed SMRT sequencing of the full-length 16S rRNA gene to obtain accurate bacterial profiles of raw milk associated with sub-clinical mastitis at the species level. A total of 21,792 circular consensus sequencing raw reads were generated from three milk samples. The Shannon index, Simpson diversity index, Chao1, and observed species of each sample were used to evaluate species richness and diversity. These values indicated that most samples exhibited a high level of bacterial biodiversity. The Shannon diversity curves indicated that the sequence depth obtained was adequate for all samples (Figure 2).

**Figure 3:**
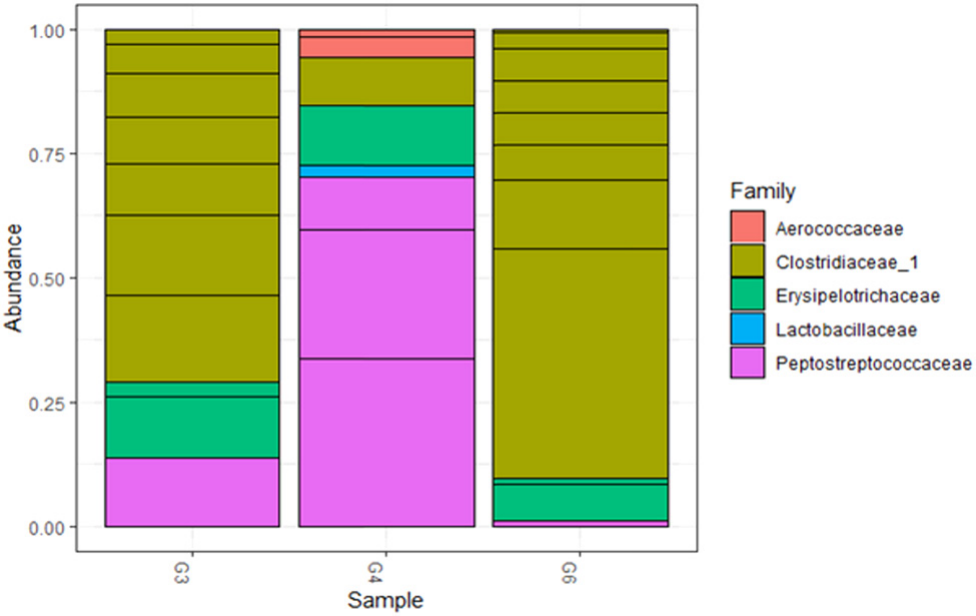
Stacked bar plot showing the top most abundant families amongst all collected samples.

**Figure 4:**
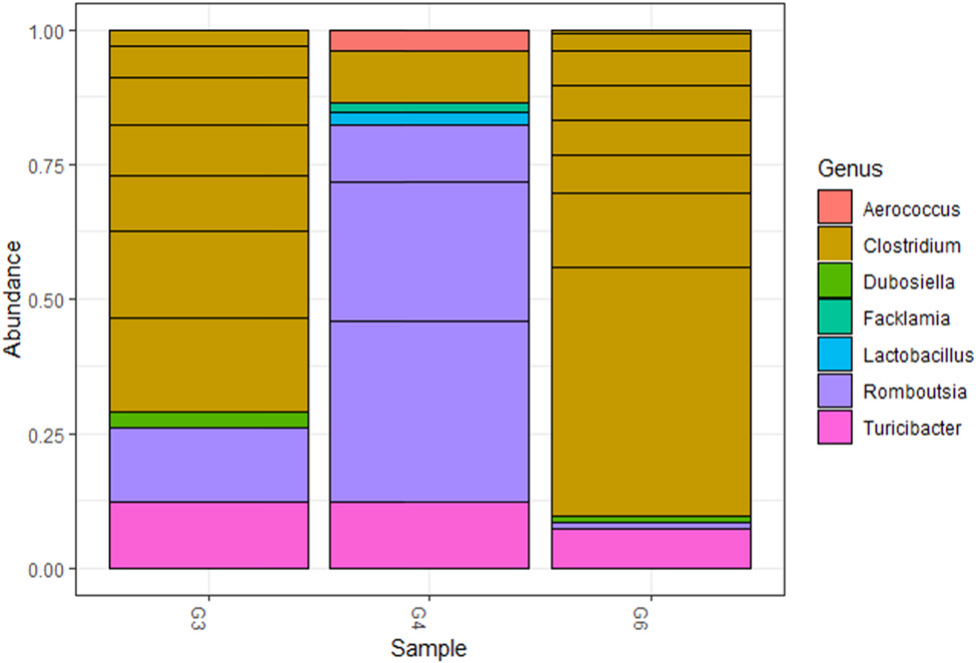
Stacked bar plot showing the top most abundant genera amongst all collected samples.

Two bacterial phyla were identified in all sampled raw milk, with Firmicutes being the most abundant, followed by Actinobacteria (Figure S2). Five families were identified, with Clostridiaceae being the most commonly identified family, followed by Peptostreptococcaceae, Erysipelotrichaceae, Aerococcaceae, and Lactobacillaceae (Figure 3). Moreover, *Clostridium* spp. was the most abundant genus, followed by *Romboutsia* spp.*, Turicibacter* spp.*, Dubosiella* spp.*, Facklamia* spp.*, Lactobacillus* spp. and *Aerococcus* spp. (Figure 4).

A total of 131 species were detected from all three samples collectively. Additionally, analysis for each sample showed the following: Sample G3 had *Turicibacter* spp. as having the largest cluster size with 87 (1.18%) followed by *Clostridium disporicum* and Clostridiaceae bacterium at 21 (0.28%) and 19 (0.26%), respectively. Sample G4 had *Aerococcus* spp. as the dominant species with a cluster size of 249 (4.76%) followed by *Turicibacter* spp. at 102 (1.95%) and *Clostridium* spp. at 14 (0.27%). Lastly, sample G6 had *Turicibacter* spp. at 196 (2.13%) followed by *Enterococcus faecalis* and *Clostridium disporicum* at 24 (0.26%) and 22 (0.24%), respectively, (Tables S1–S3).

The significant difference in the bacterial composition among the samples was confirmed by ANOVA on an excel sheet. The variance revealed that there is a significant difference in the microbial composition of sample G3 (*p* < 0.031), while the other two samples G4 (*p* > 0.058) and G6 (*p* > 0.069) had no significant difference in the microbial composition (Tables S4–S6). To further investigate the microbial composition of collected samples, richness plots were designed to understand the bacterial diversity and richness in raw milk. The Chao1 index varied from 8 to 11 with a mean value of 9.6, while the ACE index varied considerably from 0 to 11. Comparison of the Shannon indices across all samples showed bacterial diversity from farm to farm. In some samples, the values were close to the minimum value of 1.7, while some had adequately distributed microbial communities of 2.1. The Simpson index ranged from 0.76 to 0.88 (Figure 2).

## 4 Discussion

SCM is an IMI arising either from underlying infections that are not resolved in time or new infections that arise during dry climatic periods [16]. The presence of SCM in lactating cows can also be correlated with the introduction and development of clinical mastitis. The CMT remains the diagnostic tool of choice and is used to detect clinical mastitis on farms globally.

A study by Birhanu et al. [17], which evaluated automated milk leukocyte using a differential test and CMT for detecting IMIs, found that out of the 306 cows that were lactating early and late, only 25.2 and 25.8% were infected on either one or more quarters, respectively. Such high percentages of SCM could be attributed to potential risks such as age, condition of the udder, milk yield, and parity of the cows [18]. Li et al. [19] emphasize that farmers cannot only rely on the use of CMT to screen mastitis in a dairy herd, but they also need to test the milk *in vitro* to identify etiological agents. That is because CMT did not provide an adequate test sensitivity for identifying infected quarters and cows. Hence, all lactating cows should be treated as suspects for IMI, and routine biosecurity measures should be taken. Such measures include the use of disposable hand towels or gloves when handling the teat, using buckets when stripping, using disinfected hands when milking cows with low productivity, milking only twice a day, and hand washing after handling teats or milking each cow [20].

Somatic cells are an important milk constituent, and their condition is a vital indicator of teat health and the quality of the produced milk. To better understand the role of somatic cells in dairy manufacturing processes, we need to consider factors such as the physiochemical changes that occur in milk, bacterial counts, and the health status of the cow [21]. SCC are commonly used as indicators of SCM in bovines as they usually increase during IMIs caused by bacteria. Other environmental factors as well as cow-specific factors such as age, stage of lactation, the season of the year, stress, and management of the farm also play a role in SCM infections [11].

The latter author argues that standards/limits of SCC differ among countries globally. For example, the European Union regulations and New Zealand, Canada, and United States set these standards at 4 × 10^5^, 5 × 10^5^, and 7.5 × 10^5^ cells mL^−1^, respectively. The International Dairy Federation requires a limit of 5 × 10^5^ cells mL^−1^ as the standard somatic cell count for milk and milk products. Several studies have investigated the correlation between different mastitis diagnostic tests and the number of somatic cells in milk, and they have established different thresholds for diagnosing SCM. For the purpose of this study, the three thresholds to diagnose whether the cow or the teat was infected or not are as follows: SCC of 1 × 10^5^ cells mL^−1^ or less indicated an uninfected cow; SCC of 1 × 10^5^ cells mL^−1^ to 2 × 10^5^ cells mL^−1^ would indicate that a cow had an IMI in at least one or more teats; and SCC of 2 × 10^5^ cells mL^−1^ to 5 × 10^5^ cells mL^−1^ or greater indicated that the cow was infected significantly and probably had high bacterial counts.

Various studies have reported the correlation of SCC with SCM. Björk et al. [22] showed that the prevalence of SCM regardless of the number of infected teats was 51.8%. While Sonotharan et al. [23] found an even higher prevalence of clinical mastitis in Kampala, the count was 63% at teat/quarter level, with Staphylococci being the most predominant organism. An investigation of the prevalence of SCM in lactating cows by Islam et al. [24], in Batticaloa District in Sri Lanka, found that the pervasiveness of the infection was as high as 60.7% in all the lactating cows. This high percentage of infection was attributed to age, parity, and housing systems. It is also alluded by Ashraf et al. [25] that age, parity, and housing systems play a role in the prevalence of both subclinical and clinical mastitis. They found that the prevalence of mastitis was 68.0%, with SCM accounting for the highest infections in the bovines of commercial farmers in Addis Ababa. The latter authors also highlight that factors such as breed, age, parity, and period of lactation contribute to significant differences in the prevalence of mastitis among bovines. Goli et al. [26] suggest that findings of the prevalence of SCM may differ among areas depending on the diagnostic tool used.

The results of mPCR can be compared to those of Koskinen et al. [27] who detected lower numbers of these bacteria in the milk samples when they utilized mPCR to simultaneously detect mastitis-causing pathogens: i.e., 26, 12, and 6% for *S. aureus, S. agalactiae,* and *E. coli,* respectively. Because this study sought to detect subclinical and not clinical mastitis, it is possible that the circumstance of not isolating all the species under investigation could have affected the limited detection using mPCR because there may have been no viable cells of the species under investigation [28]. A similar study by Yang et al. [29] detected one pathogen in a mPCR assay at 43.5%, while only 3.8% was due to three pathogens. Pheuktes et al. [30] also recorded similar results. Although the current study did not detect multiple species in the extracted milk DNA, the identification of these pathogens by mPCR can still be helpful to get enough information regarding the causes of mastitis so that control measures can be appropriately implemented. However, it is acknowledged that factors such as PCR inhibitors can still play a role in the detection limit of mPCR. Hence, these factors need to be identified and removed to obtain more decisive results [29]. Also, to increase the sensitivity of the mPCR assay, it is advisable that the samples be enriched to obtain enough bacterial DNA so that the pathogens can be detected [30].

The study further utilized the SMRT sequencing technique to investigate and understand the raw milk microbiota of SCM cow’s diversity and showed significant characteristics of the microbial composition of raw milk in depth. Various studies have been conducted to further understand the natural bacterial communities using other sequencing platforms; however, they are limited to the identification of microbes to genus level [31,32,33,34]. Hence, third-generation sequencing such as PacBio SMRT sequencing was introduced. In the current study, PacBio SMRT sequencing technology was utilized because of its advantageous benefits over other sequencing platforms, including its capacity to generate longer reads or full-length sequence reads. In addition, the resolution from the SMRT sequencing approach enabled the detection of microbes from the higher taxonomic resolution to species level.

This study has revealed and recorded bacteria that are of importance in food microbiology. However, these bacteria are mostly not isolated and detected in raw milk. The results observed were almost similar to those in ref. [35], where it was observed that in raw milk, the most abundant taxa were Clostridiales and Lactobacillales in almost all the tested samples. The significance of *Clostridium* species in the food industry is mainly due to their neurotoxigenic properties. Species such as *Clostridium perfringens* in foods are also associated with this threat [36]. These species also contain a subgroup of bacteria known as Butyric acid bacteria that are known for spoilage. Von Neubeck et al.[37] stated that these bacteria are noteworthy due to their high prevalence in bovine milk.

*Clostridiale*s species detected in this study belong to the family of *Clostridium* of the phylum of Fermicutes. Species belonging to this family form spores when subjected to environmental factors, including osmotic pressure and extreme temperature changes. These species are frequently correlated with animal feces, soil, and inadequate udder hygiene, which may, in turn, contaminate the milk in a bulk tank [38,39]. Regarding the refrigeration temperatures, it has been observed that *Clostridium* species or strains can withstand temperatures of about 4°C for almost 11 days which is the average temperature used for the storage of milk in a farm [40]. In a similar study by Andrews et al. [41], it was found that milk samples from healthy cows contained *Turicibacter* spp., *Enterococcus* spp., *Aerococcus* spp., *Facklamia* spp., and *Clostridium* spp. together with other species of interest in dairy microbiology, such as *Staphylococcus* spp. The current study has also reported such species in raw milk from mastitis cows.

## 5 Conclusion

The study was undertaken to assess the prevalence and the extent of SCM-causing pathogens on smallholding farms in the Maluti-A-Phofung Local Municipality in the vicinity of Harrismith. Upon visiting the selected farms, it was observed that the employees used their hands to clean the udders and milk the dairy cows. It may be argued that this practice may have resulted in the isolation and identification of *S. aureus* in almost all the collected samples of raw milk. The current study could not isolate all five SCM-causing agents by utilizing conventional microbiological techniques and mPCR; however, we were able to isolate, albeit to a minimal extent, *S. aureus* and *E. coli* as well as organisms of the Streptococcal species. Since the predominant isolate was *S. aureus*, it can be concluded that contagious mastitis was prevalent in the cows under investigation. This study further used the high-throughput sequencing to further understand the microbial composition and milk diversity from SCM dairy cows. By utilizing this technique, this study observed organisms that were less detected by other similar studies. For this reason, more epidemiological and cross-sectional studies should be conducted to further understand the microorganisms involved in bovine SCM cases.
